# McGill University

**Published:** 1896-10

**Authors:** 


### McGILL UNIVERSITY.

THE veterinary department reopened for the year 1896–1897 on Tuesday, September 29, 1896. The corps of instructors remains the same for the coming collegiate year, and the maintenance of the same curriculum is adhered to that has given the school an advanced place among the seats of veterinary learning.

